# Divergent Metabolic Phenotype between Two Sisters with Congenital Generalized Lipodystrophy Due to Double AGPAT2 Homozygous Mutations. A Clinical, Genetic and *In Silico* Study

**DOI:** 10.1371/journal.pone.0087173

**Published:** 2014-01-31

**Authors:** Víctor A. Cortés, Susan V. Smalley, Denisse Goldenberg, Carlos F. Lagos, María I. Hodgson, José L. Santos

**Affiliations:** 1 Department of Nutrition, Diabetes and Metabolism, School of Medicine, Pontificia Universidad Católica de Chile, Santiago, Chile; 2 Department of Endocrinology, School of Medicine, Pontificia Universidad Católica de Chile, Santiago, Chile; INSERM/UMR 1048, France

## Abstract

Congenital generalized lipodystrophy (CGL) is a rare autosomal recessive disorder characterized by extreme reduction of white adipose tissue (WAT) mass. CGL type 1 is the most frequent form and is caused by mutations in *AGPAT2*. Genetic and clinical studies were performed in two affected sisters of a Chilean family. These patients have notoriously dissimilar metabolic abnormalities that correlate with differential levels of circulating leptin and soluble leptin receptor fraction. Sequencing of *AGPAT2* exons and exon-intron boundaries revealed two homozygous mutations in both sisters. Missense mutation c.299G>A changes a conserved serine in the acyltransferase NHX4D motif of AGPAT2 (p.Ser100Asn). Intronic c.493-1G>C mutation destroy a conserved splicing site that likely leads to exon 4 skipping and deletion of whole AGPAT2 substrate binding domain. *In silico* protein modeling provided insights of the mechanisms of lack of catalytic activity owing to both mutations.

## Introduction

Congenital generalized lipodystrophy (CGL) or Berardinelli-Seip syndrome (OMIM # *608594) is an infrequent autosomal recessive disorder characterized by severe reduction of whole body white adipose tissue (WAT) mass. CGL is usually diagnosed soon after birth and affected patients develop insulin resistance, hypertriglyceridemia, hepatic steatosis and early onset diabetes mellitus [1]–[3]. Although, four different CGL syndromes have been defined [3], 95% of reported cases correspond to CGL-1 or CGL- 2 patients.

CGL-1 is caused by mutations in *AGPAT2* gene [4] and is characterized by profound reduction of subcutaneous and visceral WAT. Severe insulin resistance and reduced levels of circulating leptin and adiponectin are typical findings along with acromegaloid features, umbilical hernia and focal lytic lesions in appendicular bones [1], [3]. CGL2 is caused by mutations in *BSCL2* gene [5] and is the most severe form of generalized lipodystrophy. These patients have virtual absence of both WAT and mechanical fat depots and frequently develop mild to moderate intellectual impairment [6].

*AGPAT2* is located on chromosome 9q34 and encodes 1-acylglycerol-3-phosphate O-acyltransferase 2. This enzyme converts lysophosphatidic acid (LPA) to phosphatidic acid (PA) in the *de novo* glycerolipid synthesis pathway [7] and is mainly expressed in WAT, pancreas and liver [8]. The exact mechanisms of lipodystrophy in patients with *AGPAT2* mutations remain elusive but possibly involve impaired adipogenesis owing to abnormal lipid signaling [9], [10].

Here we report two sisters harboring double homozygous mutations in *AGPAT2* gene, that clinically illustrate the wide spectrum and variable degree of severity of metabolic abnormalities in CGL1. We also analyze the potential structural and functional impact of these mutations on AGPAT2.

## Methods

### Patients

The reported patients are sisters, born to consanguineous parents (first-degree cousins) and originate from southern Chile. All their four grandparents were born in Chile and have Spanish last names. The maternal grandmother was sister of the paternal grandfather of the patients a no other cases of CGL have been identified in their sibship. The patients were informed of the aims of this research and signed informed consents that were previously reviewed and approved by Pontificia Universidad Católica de Chile institutional research ethics committee. This investigation was conducted after Pontificia Universidad Católica de Chile and Fondo Nacional de Ciencia y Tecnología (Fondecyt) ethics committees provided their written approval for its execution. Both patients read, discuss with the corresponding author (V.A.C.) and understood the text and figures that compose this article. The patients in this manuscript have given written informed consent to publication of their case details.

### Adipokines, Cytokines, Hormones and Plasma Biochemistry

All blood biochemical and hormone determinations were carried out after 12 hours of fasting. Leptin, leptin receptor soluble fraction (LepRs) and interleuquin-6 (IL-6) were determined by ELISA (Quantikine, R&D, Minneapolis, MN). Adiponectin was determined by RIA (Millipore, MI). Thyroid hormones, testosterone, SHBG, insulin, as well as plasma glucose, cholesterol, triglycerides, creatinine, HDL cholesterol, HbA1c and transaminases were determined at the Central Laboratory of Pontificia Universidad Católica de Chile by standard clinical laboratory procedures and techniques.

### Genetic Analysis

Genomic DNA was extracted from whole blood using QIAamp DNA Blood Mini Kit (Qiagen, Hilden, Germany). All six exons and exon-intron boundaries and 5′ and 3′ UTR regions of *AGPAT2* gene were amplified by polymerase-chain reaction (primer sequences listed in Table 1). PCR products were directly sequenced (Macrogen, Korea). The resulting sequences were compared with the published reference *AGPAT2* transcript ID (ENST 00000371696) using ChromasPro 1.5 (Technelysium, Australia). Sequence variations were named in agreement with the recommendations of the Human Genome Variation Society [11]. Intron mutation analysis was performed with Human Splice Finder engine (http://www.umd.be/HSF/) that predicts the likelihood of splicing abnormalities. Natural splicing sites (5′and 3′), branching points and exon splice enhancers are assigned a consensus values. A delta Consensus Value (ΔCV) of the splice sites strength >10% is likely to determine splicing variations [12].

**Table 1:** PCR primers used for *AGPAT2* exon - intron boundaries sequencing.

AGPAT2-E1R : 5′ AAGGAGGGAAGCCCAGAAG 3′
AGAPT2-E2F: 5′- TCCCCAGCCTCCTCCACAC-3′
AGAPT2-E2R: 5′- ACAGGCAGCCCTGTGTCCTC-3′
AGPAT2-E3F 5′ GGCCTCAGTACCTCCCTCAG 3′
AGPAT2-E3R : 5′ GGAGCATGGATGATGTAGGG 3′
AGAPT2-E4F: 5′- CCACGCCAGACCCCTACATC-3′
AGAPT2-E4R: 5′- GCCCTGTCCCCAACTCAGTG-3′
AGAPT2-E5F: 5′- ACAAGGACCCATGGCAGCAG-3′
AGAPT2-E5R: 5′- CACTCATTCGCCACATGGTCA-3′
AGAPT2-E6F: 5′- AGGGAGTCCAGGGGAAGAGC-3′
AGAPT2-E6R: 5′- GGGAGCCGGACAGAGTGGTA-3′

### *In silico* Analysis of Mutant AGPAT2 Protein Functionality

Polyphen-2 v.2.2.2r398 [13] and Provean (Protein Variant Effect Analyzer) v.1.1.3 [14] were used to assess the potential biological impact of missense mutations on AGPAT2 function. Provean works in a binary way resulting in either neutral or deleterious effect of the mutation and Polyphen renders a score between 0 and 1, being 1 the highest and most damaging score.

### AGPAT2 Molecular Modeling

A three-dimensional model for human AGPAT2 was generated by comparative modeling technique with the crystal structure of Curcubita moschata glycerol-3-phosphate acyltransferase (cmGPAT) as a template. (UniProt access numbers O15120 and P10349). Sequence alignments of hAGPAT2 and cmGPAT sequences were performed with ClustalX v2.0, using the blosum62 matrix [15], [16]. Initial alignment was further manually modified to avoid gaps in critical functional residues and the protein model constructed with Modeller program as implemented in the Build Homology Model protocol available in Discovery Studio v2.1 (Accelrys Inc., San Diego, USA). Figures were rendered with Pymol v1.4 (Schrödinger, LLC).

## Results

### Case Report: Two Sisters with CGL have Markedly Divergent Metabolic Phenotype

#### Patient 1

29-years-old woman born to consanguineous parents (first degree cousins). She was born at term (39 weeks) but her weight and height at birth data are not available. Patient 1 was diagnosed with lipoatrophic diabetes, severe mixed dyslipidemia (total cholesterol = 960 mg/dl, triglycerides = 4,355 mg/dl) and fatty liver at 13-years-old. She has been treated with restriction of dietary fat and carbohydrates intake and various combinations of insulin sensitizers (rosiglitazone or metformin), DPP IV inhibitors (sitagliptin) and insulin, but glycemic and lipid control has been poor (Table 2). Before this current evaluation, no specific pharmacologic treatment was used for controlling her plasma lipids. She refers incidental appearance of yellowish maculo-papular cutaneous lesions mainly in her back and face after insulin supplementation. Because these complications she abandoned insulin therapy 4 years ago. The patient also complains of symmetric polyarthralgia of hands and knees and morning stiffness with no local inflammatory signs. Her menstrual cycles are irregular, with very long periods (years) of amenorrhea. Physical examination revealed normal height (1.6 m), low body weight (body mass index, BMI, 18.3 kg/m^2^), generalized and severe reduction of subcutaneous fat, acromegaloid features (prognathism and enlarged hands and feet), acanthosis nigricans on the neck and groins, sparse pink-yellowish small maculopapular skin lesions in the back, suggestive of eruptive xhantomas, and a small umbilical hernia. A firm nodular mass (3 cm diameter) on each calcaneus tendon was found. No visceromegaly was detected but a moderate intensity holosytolic murmur was present at mitral focus. She had advanced bilateral proliferative retinopathy, panphotocoagulation of both eyes and amaurosis of the left eye because of vitreous hemorrhage. No signs of arthritis were found. Biochemical analysis showed poorly controlled diabetes and severe mixed dyslipidemia (Table 2). Measured creatinine clearance and proteinuria were 89.7 ml/min/1.73 m^2^ and 2.79 g/24 hours, respectively (Table 2). Endocrine evaluation showed normal-low fasting insulin, normal thyroid function, reduced SHBG and normal testosterone (Table 2). Plasma leptin was severely reduced, adiponectin was undetectable but soluble leptin receptor was ∼2-fold increased relative to a group of control women (Table 2). Antibodies against plasma cyclic citrullinated peptides were negative and no signs of arthritis were detected by magnetic resonance imaging (MRI) analysis of her hands. MRI also revealed no abnormalities of intra-abdominal organs but incidental gallstones were observed data (not shown). A liver biopsy obtained during an elective cholecystectomy revealed no hepatic steatosis or any other histological abnormality. Body composition assessed by DEXA showed 12.3% of body fat and resting energy expenditure (REE) estimated by indirect calorimetry was 32.75 Kcal/Kg lean mass/day (Table 2).

**Table 2:** Biochemical, hormone, body composition and energy expenditure characterization of two sisters with CGL1.

	Patient 1	Patient 2	Reference values^1^
**Glucose (mg/dl)**	320	119	70–99
**Insulin (mUI/ml)**	2.8	15.5	3–25
**HOMA-IR**	2.21	4.54	<2.6
**Cholesterol (mg/dl)**	510	239	<200
**HDL (mg/dl)**	32	29	>40
**Triglycerides (mg/dl)**	1,683	400	<150
**SGOT (U/L)**	24	56	9–25
**SGPT (U/L)**	21	35	7–30
**Creatinine (mg/dl)**	0.68	0.51	0.5–0.9
**GFR (ml/min/1.73** **m** 2 **)**	89.7	152.6	
**Proteinuria (g/24 h)**	2.79	0.063	<0.15
**Free T4 (ng/dl)**	1.3	1.12	0.93–1.7
**TSH (µUI/ml)**	3.6	1.52	0.3–4.2
**SHBG (nmol/l)**	8.0	28.4	19.1–145
**Testosterone (ng/dl)**	28.6	62.8	5–52.2
**Leptin (ng/ml)**	0.32	1.27	3.8–68.2^2^
**Adiponectin (mg/ml)**	N.D.	N.D.	7.34–24.3^2^
**LepRs (ng/ml)**	93.35	44.7	35–52.9^2^
**Body fat (%)**	12.3	11.6	
**Resting energy expenditure (Kcal/Kg lean mass/day)** 3	32.75	25.97	N.D.

1 Reference values of normal Chilean female population (Pontificia Universidad Católica de Chile).

2 Reference values obtained from 13 healthy subjects matched by age and sex.

3 Reference resting energy expenditure was individually determined by instrument’s internal algorithm on the basis of anthropometry, age and sex individual’s data.

Abbreviations: A1C, glycated hemoglobin; HOMA-IR, HOMA-insulin resistance; HDL, high density lipoprotein, SGOT, serum glutamic oxalacetic transaminase; SGPT, Serum glutamic pyruvic transaminase; Free T4, free thyroxine; TSH, thyroid stimulating hormone; SHBG, sex hormone binding globulin; LepRs, leptin receptor soluble fraction; N.D., not-detected.

#### Patient 2

26-years-old woman, sister of patient 1. She was born at preterm (36 weeks) but her weight and height data at birth are unavailable. Patient 2 was diagnosed with hypertriglyceridemia when she was 5-years-old and hyperglycemia when she was 19-years-old. Originally, this patient’s disease was recognized as type 2 diabetes mellitus and was treated with restriction of dietary fats and carbohydrates, and submaximal doses of metformin. Lipodystrophy was not previously diagnosed although she refers a persistent and marked lean phenotype. Her menarche was at 12 years old and evolved with irregular menstruations. Polycystic ovary syndrome was diagnosed at 14 years old and received estrogen-based oral contraceptives. This therapy was associated with a severe increase in plasma triglycerides and appearance of maculopapular skin lesions that were diagnosed as eruptive xhantomatosis. These complications lead her to abandon oral contraceptives. No other abnormalities were referred by the patient. Physical examination revealed severe reduction of whole body subcutaneous WAT and acromegaloid features. Biochemical blood analysis revealed diabetes mellitus, mixed dyslipidemia and mild elevation of SGOT and SGPT levels (Table 2). Measured creatinine clearance and proteinuria were 152.6 ml/min/1.73 m^2^ and 0.063 g/24 hours, respectively (Table 2). Hormone analysis showed normal thyroid function, moderately elevated testosterone and normal SHBG (Table 2). Plasma leptin, although higher than in patient 1, was lower and soluble leptin receptor was normal in comparison with a group of control women (Table 2). Adiponectin remained undetectable (Table 2). MRI revealed no abnormalities of intra-abdominal organs (data not shown). Body composition assessed by DEXA showed 11.6% of body fat and REE estimated by indirect calorimetry was 25.97 Kcal/Kg lean mass/day (Table 2).

During the preparation of the manuscript, patient 2 coursed an uncomplicated pregnancy and was able to vaginally deliver a full-term, phenotypically normal, 3,415 g female fetus. Glycemic control during her pregnancy was achieved with restriction of dietary carbohydrates and metformin 1,700 mg/day. Insulin (10 units of NPH insulin/day) was used only the last two weeks of her pregnancy. At the time of delivery, her HbA1c was 6.7% and her fasting glucose was 115 mg/dl.

### Genetic Analysis

**Figure 1:**
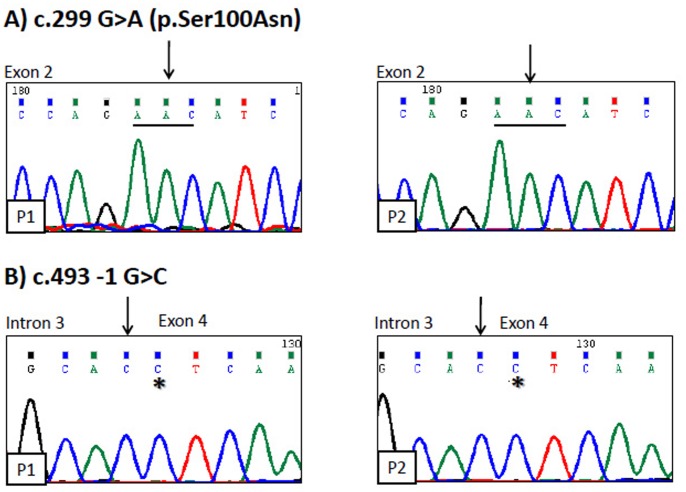
*AGPAT2* is double mutated in two sisters with CGL. **A)** Patient 1 (P1) and patient 2 (P2) harbor the single nucleotide substitution c.299G>A in exon 2 of *AGPAT2* gene (black arrows). This changes wild type codon AGC (not shown), that encodes for serine, to AAC (underlined), that encodes for Asparagine. **B)** Patient 1 (P1) and patient 2 (P2) also harbor the single nucleotide substitution c.493-1G>C (black arrows) in intron 3– exon 4 boundary. *denotes the first nucleotide of exon 4.

Clinical features strongly suggested the diagnosis of CGL1 in both patients; thus, AGPAT2 exons and exon-intron boundaries were sequenced. As shown in Figure 1A, both sisters harbor a homozygous G for A substitution at position c.299 of AGPAT2 cDNA (c.299G>A). This mutation changes an evolutionary conserved serine for asparagine in the acyltransferase NHX4D motif of AGPAT2 (p.Ser100Asn).

The potential impact of p.Ser100Asn mutation on AGPAT2 protein function was assessed with Polyphen [13] and Provean [14] algorithms. Polyphen analysis determined that this mutation was “probably damaging” with a score of 0.995, whereas Provean described it as “deleterious”.

A second homozygous mutation, G for C substitution at position −1 of intron 3 (c.493-1G>C), was also found in both patients (Fig. 1B).

As expected, patients’ parents were heterozygous for these two mutations and these sequence variants were absent in 50 unrelated control subjects (data not shown), confirming their pathogenic role in the patients.

Intronic substitution c.493-1G>C is located in a highly conserved 3′ splicing acceptor site. It has been shown that equivalent alterations determine pre-mRNA processing abnormalities, often resulting in exon skipping [17]. Thus, on-line software Human Splicing Finder (HSF) was used to evaluate AGPAT2 c.493-1G>C variant. This analysis revealed a ΔCV of −31% (CV wild type = 93.26 vs CV mutant = 64.31) in the strength of the 3′ acceptor splicing site. In this algorithm, a ΔCV larger than 10% is considered predictive of splicing variations [12]. MaxEnt, a second HSF algorithm, showed a −81.4% variation in the splice site strength, also suggesting a faulty splicing of exon 4 during AGPAT2 pre-mRNA processing. Thus, c.493-1G>C mutation possibly results in AGPAT2 exon 4 skipping [17]. Importantly, since the absence of exon 4 does not shift AGPAT2 mRNA reading frame, it should generate a protein product 246 amino acids shorter than wild type AGPAT2 variant, lacking the entire segment between p.165Leu and p.196Gln residues. Importantly, this segment includes the conserved PEGTR domain, which has been recognized essential for acyl-CoA transferase activity in all known members of this family [18].

### *In silico* Modeling of Human AGPAT2

**Figure 2:**
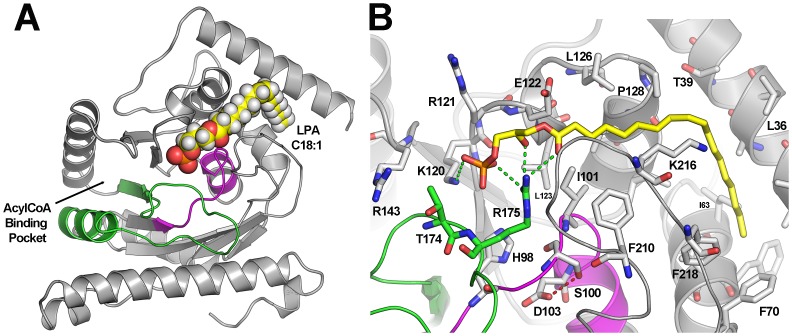
Molecular modeling of hAGPAT2. **A**) Representation of the secondary structure of the modeled human AGPAT2 protein. Green color denotes the segment between Leu165 and Ala195 that contains the acyl-glycerol-3-phosphate substrate binding motif (PEGTR) and that is encoed by exon 4. This segment is possibly deleted in c.493-1G>C mutants and converge with the catalytic site, that is colored in magenta. **B)** Snapshot of the proposed molecular interactions between LPA (C18∶1) and human AGPAT2. The polar part of the substrate make contacts with a cluster of basic residues, while its alkyl chain is accommodated inside a tunnel made of a cluster of hydrophobic aminoacid side chains.

Human AGPAT2 was modeled with cmGPAT crystal structure as template. Figure 2 shows the proposed structure of human AGPAT2 and its interaction with LPA (C18∶1). As expected, the AGPAT2 model resembles other GPAT topologies, with a packed domain composed of 12 alpha helices and 7 beta sheets (Figure 2A). In this model, the polar head of LPA makes polar contact with residues p.175Arg and p.120Lys, whereas the bulky acyl chain of LPA is accommodated within a hydrophobic tunnel made of the side chains of p.101Ile, p.210Phe, p.122Glu, p.126Leu, p.39Thr, p.36Leu, p.63Ile, p.218Phe and p.50Phe. An internal hydrogen bond occurs between p.100Ser and p.103Asp in the central section of the conserved NHX4D motif, which stabilize the turn after this helix (Figure 2B). Importantly, neither pSer100N substitution nor p.165Leu to p.196Gln deletion was expected to change AGPAT2 folding (data not shown).

## Discussion

This is the first report of Chilean patients with congenital generalized lipodystrophy. Chilean population is genetically heterogeneous, with contribution of various Amerindian and northern hemisphere people, mainly southern Europeans (Spanish and Italians) but also northern Europeans (Germans) and Middle-Easterners (Palestinians and Syrians). Interestingly, previously reported individuals with mutations in *AGPAT2* gene mainly originated from sub-Saharan Africa, Middle East and Northern Europe [19]. All the known ancestors of patients reported here were born in Chile and have Spanish last names, suggesting that they have a genetic heterogeneity level analogous to the rest of the Chilean population.

Clinically, these cases illustrate the wide spectrum of metabolic abnormalities that can be associated with severe reduction of body adiposity. However, it is puzzling that whereas the two sisters reported here share identical abnormalities in *AGPAT2* gene and equivalent level of lipodystrophy, they have notoriously dissimilar severity in their metabolic complications. In fact, whereas patient 1 has developed severe blood glucose and lipid dysregulation, advanced retinal and renal diabetic microvascular complications and secondary amenorrhea, patient 2 has only mild to moderate metabolic dysregulation and was able to course a near physiologic pregnancy. The factors and mechanisms underlying these differences remain unknown. Nonetheless, it is noteworthy that total circulating leptin levels are lower and soluble leptin receptor levels are higher in the subject with the most severe phenotype (patient 1). We speculate that differences in free circulating leptin could determine clinically significant divergent metabolic phenotype among these patients with CGL1. It is also notorious that, in contrast to previously reported CGL cases, patient 1 has no evidence of fatty liver disease. We propose that this might result from the combined effect of low dietary fat intake and low insulin driven hepatic *de novo* lipogenesis.

From the genetic stand point, it is also puzzling that two potentially deleterious mutations coexist in the same gene. This combined mutational state was also noted in a CGL patient original from Argentina [20]. A European CGL patient with c.299G>A mutation was reported by Magré et al [21], however, the second mutation (c.493-1G>C) was not reported in this subject.

The mechanisms of lipodystrophy in patients with mutation of *AGPAT2* gene remain unknown. Since AGPATs are critical for the biosynthesis of both glycerophospholipids and triglycerides, it is logical to assume that loss of fat might be the consequence of decreased synthesis of these lipid products. However, several facts indicate that this simple hypothesis may not be fully explanatory. First, the two major AGPAT isoforms (1 and 2) are expressed in mature white adipocytes and, when are co-expressed in tagged forms in cultured cells, both co-localize in the same subdomains of the endoplasmic reticulum [8]. Furthermore, AGPAT1 and AGPAT2 catalytic activities are very similar in terms of substrates preference and kinetic properties [8]. Thus, it remains unclear why AGPAT1 does not compensate AGPAT2 loss in adipocytes. Many reported mutations in *AGPAT2* gene result in reduced AGPAT2 protein and/or activity levels [22], [23], suggesting that AGPAT activity is necessary for normal WAT mass. Indeed, total loss of AGPAT2 results in a severe CGL phenotype in mice [24], [25]. The prevailing hypothesis is that AGPAT2 is required for normal adipogenesis likely by generating normal lipid signaling molecules required for adipocytes differentiation [10].

The substitution of G for C in position −1 of intron 3 (c.493-1G>C) might result in deletion of whole exon 4 due to abnormal splicing of AGPAT2 pre-mRNA. In fact, analysis with two HSF algorithms predicted that this mutation should determine high likelihood of splicing faults. Although this prediction has not been experimentally tested yet, it might result in the in-frame deletion of 32 aminoacids, including the highly conserved PEGTR motif, which is present in GPAT, AGPAT, DHAPAT and LPEAT enzymes [26].

PEGTR motif is crucial for acyltransferase catalytic activity [18]. Indeed, Arg175 has been shown to be necessary for the binding of the charged portion of *sn*-glycerol-3-phosphate substrates in this family of enzymes [26], [27]. Therefore, although the deletion of the entire Leu165 - Ala195 segment should not affect global AGPAT2 folding it might severely affect substrate binding, resulting in loss of catalytic activity.

The other mutation observed in the reported patients (c.299G>A), might additionally affects enzyme catalysis in two different ways. First, it precludes the formation of an intramolecular hydrogen bond between Ser100 and the conserved Asp103, which is important for the charge relay system for the conserved catalytic His98 [26]. Second, the bulkier Asn side-chain might increases the steric hindrance of the catalytic pocket, possibly disrupting its topology and reducing its acyltransferase activity, such as been previously reported for bacteria [28].

Functional and biochemical studies will be required to empirically evaluate the effects of these mutations on AGPAT2 enzymatic activity as well as its effects on adipogenesis.

In summary, we have reported the first two cases co CGL in Chile. These sisters harbor identical two homozygous single nucleotide substitutions in *AGPAT2* gene, have severe reduction of body adiposity but have developed markedly dissimilar metabolic phenotype, possibly as a result of different residual leptin levels. Structurally, c.493-1G>C mutation potentially results in loss of the highly conserved substrate binding PEGTR domain of AGPAT2 protein, whereas c.299G>A mutation results in the substitution of a highly conserved serine in the catalytic NHX4D motif of the protein.
